# Reduction in social anxiety after MDMA-assisted psychotherapy with autistic adults: a randomized, double-blind, placebo-controlled pilot study

**DOI:** 10.1007/s00213-018-5010-9

**Published:** 2018-09-08

**Authors:** Alicia L. Danforth, Charles S. Grob, Christopher Struble, Allison A. Feduccia, Nick Walker, Lisa Jerome, Berra Yazar-Klosinski, Amy Emerson

**Affiliations:** 1 Los Angeles Biomedical Research Institute, Harbor-UCLA Medical Center, Box 498, 1000 West Carson Blvd., Torrance, CA 90509 USA; 2 Department of Psychiatry, Harbor-UCLA Medical Center, Box 498, 1000 West Carson Blvd., Torrance, CA 90509 USA; 3 MAPS Public Benefit Corporation, 1115 Mission Street, Santa Cruz, CA 95060 USA; 4 School of Undergraduate Studies, California Institute of Integral Studies, 1453 Mission Street, San Francisco, CA 94103 USA; 5 Multidisciplinary Association for Psychedelic Studies, 1115 Mission Street, Santa Cruz, CA 95060 USA

**Keywords:** Social anxiety, MDMA, 3,4-Methylenedioxymethamphetamine, MDMA-assisted psychotherapy, Autism, Asperger’s, Liebowitz Social Anxiety Scale, Psychedelics, Anxiety

## Abstract

**Rationale:**

Standard therapeutic approaches to reduce social anxiety in autistic adults have limited effectiveness. Since 3,4-methylenedioxymethamphetamine (MDMA)-assisted psychotherapy shows promise as a treatment for other anxiety disorders, a blinded, placebo-controlled pilot study was conducted.

**Objectives:**

To explore feasibility and safety of MDMA-assisted psychotherapy for reduction of social fear and avoidance that are common in the autistic population.

**Methods:**

Autistic adults with marked to very severe social anxiety were randomized to receive MDMA (75 to 125 mg, *n* = 8) or inactive placebo (0 mg, *n* = 4) during two 8-h psychotherapy sessions (experimental sessions) in a controlled clinical setting. Double-blinded experimental sessions were spaced approximately 1 month apart with 3 non-drug psychotherapy sessions following each. The primary outcome was change in Leibowitz Social Anxiety Scale (LSAS) Total scores from Baseline to one month after the second experimental session. Outcomes were measured again six months after the last experimental session.

**Results:**

Improvement in LSAS scores from baseline to the primary endpoint was significantly greater for MDMA group compared to the placebo group (*P* = 0.037), and placebo-subtracted Cohen’s *d* effect size was very large (*d* = 1.4, CI − 0.074, 2.874). Change in LSAS scores from baseline to 6-month follow-up showed similar positive results (*P* = 0.036), with a Cohen’s *d* effect size of 1.1 (CI − 0.307, 2.527). Social anxiety remained the same or continued to improve slightly for most participants in the MDMA group after completing the active treatment phase.

**Conclusions:**

This pilot trial demonstrated rapid and durable improvement in social anxiety symptoms in autistic adults following MDMA-assisted psychotherapy. Initial safety and efficacy outcomes support expansion of research into larger samples to further investigate this novel treatment for social anxiety.

**Trial registration:**

clinicaltrials.gov identifier, NCT02008396

**Electronic supplementary material:**

The online version of this article (10.1007/s00213-018-5010-9) contains supplementary material, which is available to authorized users.

## Introduction

In humans, 3,4-methylenedioxymethamphetamine (MDMA) generates feelings of social affiliation and increases social approach while diminishing negative responses to social rejection (Kamilar-Britt and Bedi 2015). “Ecstasy” or “molly” refers to chemical entities represented as containing MDMA. MDMA is primarily a potent releaser of serotonin and norepinephrine, and to a lesser extent dopamine (de la Torre et al. 2004; Hysek and Liechti 2012). MDMA also promotes release of the neurohormone oxytocin (OT) (Dumont et al. 2009; Hysek et al. 2012; Kirkpatrick et al. 2014; Kuypers et al. 2017). OT is associated with social affiliation in mammals and attenuates amygdalar response to anxiogenic stimuli (Adolphs et al. 2005; Bartz and Hollander 2006), and OT receptor gene variations may also modulate prosocial effects of MDMA in humans (Bershad et al. 2016; Vizeli and Liechti 2018).

Due to its unique pharmacology, MDMA has shown promise as an adjunct to psychotherapy for treatment of posttraumatic stress disorder (Mithoefer et al. 2018; Mithoefer et al. 2011; Mithoefer et al. 2013; Oehen et al. 2013). Anticipating concerns about using a schedule 1 substance in a clinical trial with an autistic adult population, we published a preliminary paper on study rationale and methods including information on history, pharmacology, effects in animals and humans, safety, and clinical advantages of MDMA (Danforth et al. 2016).

*Autism* refers to a spectrum of congenital and pervasive neurocognitive variants. Autism presents with myriad manifestations resulting in considerable heterogeneity among individuals with atypical development of social and communication skills. At present, there are no published research data in support of compounds that can influence the course of autism or be a causative agent (Danforth 2013). There may be underlying biological reasons autistic adults have atypical responses to psychiatric medications commonly prescribed for anxiety, including evidence for fewer benzodiazepine binding sites, atypical GABAergic inhibitory signaling, and atypical serotonin and dopamine transporter binding in autistic brains (Coghlan et al. 2012; King et al. 2009; Nakamura et al. 2010; Uzunova et al. 2016).

Qualitative data on MDMA/ecstasy use by autistic adults in epidemiological settings supported the selection of *social anxiety disorder* (SAD) as the primary indication for this study (Danforth 2013). SAD is characterized by fear of scrutiny and avoidance of social interactions (American Psychiatric Association 2013). Comparative studies suggest that autistic individuals are at greater risk (1:4) of current or lifetime SAD (Bejerot et al. 2014). The study presented here is the first controlled study of MDMA-assisted psychotherapy in autistic adults. The objective of this investigational treatment was not to cure or alter the course of autism but to explore the feasibility and safety of treating SAD with MDMA-assisted psychotherapy in this underserved population.

## Methods

### Trial design

We employed a randomized, placebo-controlled, double-blind methodology for this exploratory phase 2 single-site study conducted from February 2014 through April 2017. The authors assert that all procedures contributing to this work comply with ethical standards of relevant national and institutional committees on human experimentation and with the Helsinki Declaration of 1975, as revised in 2008. The study was approved by Los Angeles BioMedical Research Institute IRB and conducted in accordance with Good Clinical Practice. Twelve participants were enrolled and randomized to receive MDMA (*n* = 8) or inactive placebo (*n* = 4). MDMA was synthesized by David Nichols at Purdue University, compounded with lactose, and placed into gelatin capsules by a research pharmacist. The inactive placebo, lactose, was filled in equivalent weight in identical capsules. After three 60- to 90-min non-drug preparatory psychotherapy sessions, participants received two blinded experimental sessions with MDMA or placebo, spaced approximately 1 month apart. Following each experimental session, three 60- to 90-min non-drug integrative psychotherapy sessions occurred over 3 weeks. The blind was broken at 6-month follow-up; participants who received placebo in the first treatment phase returned for two optional open-label treatment sessions with MDMA (data not presented).

A dose-finding study design was selected in response to anecdotal data, suggesting that hyper-reactivity to sensory stimulation and emotion regulation challenges associated with autism might indicate the need for a lower, yet therapeutically active, MDMA dose range. Among participants receiving MDMA, the first subgroup (*N* = 4) received 75mg MDMA at the first session and 100-mg MDMA at the second session. The second subgroup (*N* = 4) received 100mg MDMA at the first session and 125 mg at the second session. All doses were tolerated well; no participants declined the option to escalate the dose for the second session.

An independent rater (IR) administered the Leibowitz Social Anxiety Scale (LSAS) (Liebowitz et al. 1985) at baseline, 1 day, 2 weeks, and 4 weeks after each experimental session and re-administered it before the blind was broken at 6 months. There was one LSAS IR for the entire study to minimize variance. The primary outcome was change from baseline to 1-month post second experimental session in LSAS total scores. At monthly intervals, between the 1-month post-treatment psychotherapy session and the 6-month follow-up visit, participants completed the Beck Depression Inventory (BDI-II) (Beck et al. 1996), Spielberger State-Trait Inventory (STAI Form Y-2) (Spielberger et al. 1983), and Perceived Stress Scale (PSS) (Cohen et al. 1983) through an electronic Patient Reported Outcome (ePRO) system (Medrio, CA, USA).

### Screening, eligibility, and participants

Participants were recruited through Internet advertisements, word of mouth, and clinician referrals. No participants in this study were under conservatorship; all signed an informed consent after review with investigators. Eligibility was established through clinical interview and administration of diagnostic instruments, including the Structured Clinical Interview for Diagnostic and Statistical Manual of Mental Disorders- Fourth Edition Axis I Research Version (SCID-I-RV) (First et al. 2002), the Columbia Suicide Severity Rating Scale (C-SSRS) (Posner et al. 2011; Posner et al. 2007), LSAS (Liebowitz et al. 1985), and the Autism Diagnostic Observation Schedule (ADOS-2 Module 4) (Bastiaansen et al. 2011). To be eligible, a global LSAS score of 60 or higher, indicating marked to severe fear and avoidance of specific social situations, was required. Participants were 21 or older, MDMA naïve by self-report, physically healthy, and psychologically stable (see Supplemental for inclusion/exclusion criteria).

### Preparatory and integrative psychotherapy

All participants received three preparatory psychotherapy sessions, during which past or current salient issues in the participant’s life were discussed. These sessions focused on establishing rapport between the participant and treatment team. In two instances, an additional preparatory session was required to accommodate clinical considerations.

Research findings support mindfulness-based therapies for autistic adults (Spek et al. 2013). Consequently, participants received standardized mindfulness-based therapy adapted from dialectical behavioral therapy (DBT) as part of their treatment (Linehan 1993). DBT was developed to support individuals struggling with interpersonal relationships, emotion regulation, and distress tolerance. In general, these psychosocial domains are challenging for autistic adults with SAD. A notable advantage of mindfulness-based preparatory psychotherapy was the introduction of vocabulary and skills that helped participants with transitioning into MDMA-influenced cognitive and affective states, as well as with communicating with others during novel, often ineffable, altered states of consciousness.

### Experimental sessions

For experimental sessions, participants arrived around 09:30. They were required to refrain from eating after 24:00 (midnight) except for non-alcoholic fluids prior to the session. Study visits took place in a room with a den-like ambiance, which was designed to minimize sensory distress (e.g., soft lighting, noise abatement). Per consultation with members of the autistic community, features such as elements of nature (e.g., fresh flowers), “fidget” objects for self-regulating through repetitive movement (“stimming”), and suitable décor items were added to support common autistic preferences. Additionally, the room accommodated esthetic adjustments for comfort (e.g., seating arrangements, temperature) and had an adjacent private lavatory.

Study drug was administered around 10:30 after a guided progressive muscle relaxation exercise (McCallie et al. 2006). The experimental sessions were video-recorded, contingent upon participant consent for adherence rating and training purposes. Water intake was monitored to avoid dehydration or water intoxication; optional snacks and a light meal were made available 3 h after study drug administration. Sessions concluded in late afternoon, and participants were ready to leave around 17:30 after a brief closing. Participants were given a contact for urgent assistance from an investigator physician.

### Post-session follow-up

The morning following each experimental session, participants returned to the center for integrative psychotherapy. Safety data were collected, the content of the previous day’s experience was examined, and methods for adjusting back to daily life after treatment were reviewed. Two in-person integrative psychotherapy sessions were scheduled at 2-week intervals for 1 month and again at the 6-month follow-up point, with the option of adding an additional office visit, if needed. Telephone safety checks occurred each of the 7 days following experimental sessions. During these calls, spontaneously reported reactions were recorded; the call length was extended to provide additional time for processing cognitive and affective responses, as appropriate.

### Assessments

The primary outcome measure was the LSAS, a 24-item, semi-structured interview evaluating the severity of social anxiety symptoms. The LSAS has been used widely in studies, including research on SAD in autistic adults (Bejerot et al. 2014). In addition, change from baseline was assessed with secondary measures, including BDI-II, PSS, Interpersonal Reactivity Index (IRI) (Davis 1980; Davis 1983), Rosenberg Self-Esteem Scale (RSES) (Rosenberg 1965), STAI, Toronto Alexithymia Scale (TAS-20) (Bagby et al. 1994), The Awareness of Social Inference Test (TASIT) (McDonald et al. 2006), and Emotion Regulation Questionnaire (ERQ) (Gross and John 2003).

### Pharmacodynamic measures

Blood pressure, heart rate (GE Medical Systems Information Technologies, Tampa, FL), and temperature (Braun, Kronberg im Taunus, Germany) monitoring was performed pre-drug, then hourly for 6 to 7 h following administration of active drug or placebo.

### Safety monitoring

Investigators collected adverse events and concomitant medications at each visit and spontaneously reported reactions during experimental sessions and 7 days after. Suicidal ideation was assessed with the C-SSRS at the beginning and end of treatment days; subjective units of distress (SUDs) were assessed hourly to determine need for additional support. In addition, a consented study support partner (SSP) drove the participant to and from experimental sessions and from day-after integrative sessions. The participant could choose any trusted adult as their SSP. SSPs were instructed to serve as a nonintrusive supportive presence and to remain in the same location or close by to the participant after treatment through the visit the next morning.

### Statistical analysis

Statistical Package for the Social Sciences (SPSS), version 20 (IBM Corp, Chicago, IL), was used for analyses. Data from the MDMA dose subgroups were combined into one MDMA group (75–125 mg) for analysis due to the small sample size and all doses being within the active therapeutic range of MDMA. Independent samples *t* tests were used to test for significant changes in LSAS total score from baseline to 1 month post-second experimental session, designated as the primary endpoint, and from baseline to 6-month follow-up. Analyses of secondary outcome measures were exploratory; descriptive statistics are presented. The alpha level indicating significance for primary analysis was 0.05 (two-tailed). Effect sizes were estimated using Cohen’s *d* independent-groups pretest-post-test design (Kadel and Kip 2012). Primary outcome results from one participant in the MDMA group are missing due to emerging medical history information, which indicated that the participant no longer satisfied inclusion criteria. For the intent-to-treat set, this participant’s baseline scores were included; missing data was not imputed.

## Results

### Demographics

**Fig. 1:**
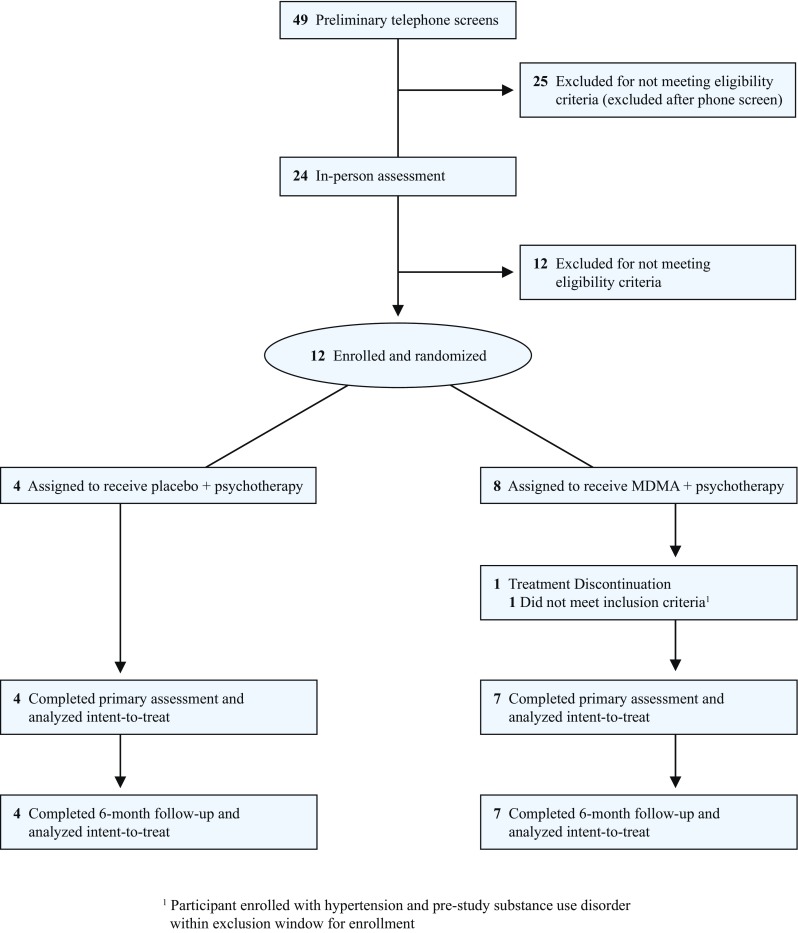
CONSORT diagram

Recruitment occurred from 2014 to 2016; all planned participants were enrolled; study visits were completed from 2014 to 2017. Forty-nine participants were evaluated for eligibility by telephone; 24 were further assessed in person; of these, 12 were enrolled and randomized (Fig. 1). Participants were 31.3 (SD: 8.8) years old on average and identified as 83.3% male and 16.7% female, with all females being randomized to the MDMA group. Despite a small sample size, ethnic backgrounds of participants were reasonably diverse, and 25% reported non-heteronormative sexual orientation. Mean baseline BMI was greater in the placebo group than the MDMA group. The majority of participants had previously received psychotherapy, primarily supportive talk therapy (83.3%). Eight of 12 (66.7%) participants had received pharmacologic treatments, primarily antidepressants (58.3%), stimulants (33.3%), and anxiolytics (25.0%). Baseline LSAS ratings ranged from 69 to 125, indicating marked to very severe SAD symptoms. Based on medical history and confirmed with the SCID, 66.67% of participants had a history of depression, 41.67% had generalized anxiety disorder, and 100% had SAD. Two participants had exhibited past suicidal behavior, one participant had past serious ideation, and seven exhibited positive suicidal ideation based on the Lifetime C-SSRS. See summary of participant demographics (Table 1) and baseline characteristics (Table 2).

**Table 1:** Demographics

	Placebo (*n* = 4)	MDMA (*n* = 8)	Total (*n* = 12)
Age, mean (SD), y	28.3 (3.8)	32.8 (10.4)	31.3 (8.8)
Sex, no. (%)			
Male	4 (100.0)	6 (75.0)	10 (83.3)
Female	0	2 (25.0)	2 (16.7)
Ethnicity, no. (%)			
White/Caucasian	2 (50.0)	4 (50.0)	6 (50.0)
Latino/Hispanic	0	2 (25.0)	2 (16.7)
Asian/Pacific Islander	1 (25.0)	0	1 (8.3)
Middle Eastern	1 (25.0)	0	1 (8.3)
Asian & Caucasian	0	1 (12.5)	1 (8.3)
Hispanic & Caucasian	0	1 (12.5)	1 (8.3)
BMI, mean (SD)	28.8 (9.7)	25.7 (4.3)	26.7 (6.3)
Employment status, no. (%)			
Full-time employment	0	4 (50.0)	4 (33.3)
Part-time employment	2 (50.0)	0	2 (16.7)
Student	1 (25.0)	1 (12.5)^a^	2 (16.7)
Unemployed	1 (25.0)	3 (37.5)	4 (33.3)

Abbreviations: *N*, number of participants

^a^Student with part-time employment

**Table 2:** Baseline characteristics

	Placebo (*n* = 4)	MDMA (*n* = 8)	Total (*n* = 12)
Previous psychotherapy, no. (%)^a^			
Psychodynamic	3 (75.0)	7 (87.5)	10 (83.3)
Cognitive processing therapy	1 (25.0)	0	1 (8.3)
Other	1 (25.0)	5 (62.5)	6 (50.0)
Pre-study psychiatric medications, no. (%)			
Antidepressants	2 (50.0)	5 (62.5)	7 (58.3)
Anxiolytics	0	3 (37.5)	3 (25.0)
Antipsychotics	1 (25.0)	1 (12.5)	2 (16.6)
Sleep aids	0	0	0
Stimulants	1 (25.0)	3 (37.5)	4 (33.3)
Other	0	3 (37.5)	2 (16.6)
Psychiatric comorbid disorders, no. (%)			
Major depression	1 (25.0)	3 (37.5)	4 (33.3)
Depression	1 (25.0)	3 (37.5)	4 (33.3)
Anxiety	0	4 (50.0)	4 (33.3)
Acute stress disorder	1 (25.0)	0	1 (8.3)
Generalized anxiety	0	2 (25.0)	2 (16.6)
Panic disorder	0	1 (12.5)	1 (8.3)
Obsessive compulsive disorder	1 (25.0)	1 (12.5)	2 (16.6)
Attention deficit/hyperactivity disorder	1 (25.0)	2 (25.0)	3 (25.0)
Affective disorder	1 (25.0)	0	1 (8.3)
Personality disorder	0	1 (12.5)	1 (8.3)
Polysubstance dependence	0	1 (12.5)	1 (8.3)
Alcohol abuse	0	1 (12.5)	1 (8.3)
Substance use	1 (25.0)	1 (12.5)	2 (16.6)
Posttraumatic stress disorder	1 (25.0)	2 (25.0)	3 (25.0)
Lifetime C-SSRS^b^			
Positive ideation	3 (75.0)	4 (50.0)	7 (58.3)
Serious ideation	0 (0)	1 (12.5)	1 (8.3)
Positive behavior	1 (25.0)	1 (12.5)	2 (16.6)
Baseline C-SSRS^b, c^			
Positive ideation	0 (0)	0 (0)	0 (0)
Serious ideation	0 (0)	0 (0)	0 (0)
Positive behavior	0 (0)	0 (0)	0 (0)

Abbreviations: PI, positive ideation; SI, serious ideation; PB, positive behavior; *N*, number of participants

^a^Previous psychotherapy “other” included: acceptance and commitment, behavioral coaching, cognitive behavioral analysis, dialectical behavior therapy (DBT)-informed, eye movement desensitization and reprocessing (EMDR), family therapy, group therapy, and neurofeedback

^b^According to the C-SSRS scoring guide, scores of four or five on the suicidal ideation category are considered serious ideation, and scores of one or greater are considered positive behavior or ideation

^c^Baseline represents measures taken during preparatory sessions and before drug administration in experimental session 1

### Clinical response

Reduction in SAD symptoms (Table 3) as indicated by mean change in LSAS score from baseline to primary endpoint was significantly greater for the MDMA group than for the placebo group (*t*(9) = 2.451, *P* = 0.037, CI 1.92, 47.87). The placebo-subtracted Cohen’s *d* effect size was 1.4 (CI − 0.074, 2.874). At 6-month follow-up, the decline in mean LSAS score from baseline was largest for the MDMA group compared to placebo group (*t*(9) = 2.454, *P* = 0.036, CI 1.92, 47.01). The placebo-subtracted Cohen’s *d* effect size was 1.1 (CI − 0.31, 2.53). Mean (SD) LSAS scores changed minimally from primary endpoint to 6-month follow-up for both groups [MDMA 46.4 (15.2) to 42.9 (20.4), placebo 64.0 (13.3) to 60.0 (17.4)].

**Table 3:** Liebowitz social anxiety total scores, severity categorization and changes in total score^a^

	Placebo (*n* = 4)		MDMA (*n* = 8)^c^
Primary efficacy variable LSAS total score, mean (SD)			
Baseline	83.3 (11.9)		91.8 (15.8)
Primary endpoint	64.0 (13.3)		46.4 (15.2)
Change^b^	− 19.3 (18.8)		− 44.1 (14.8)
*P* value^b^		0.037	
Primary endpoint, no. (%) LSAS 20-point reduction			
Yes	2 (50.0)		6 (85.7)
No	2 (50.0)		1 (14.3)
LSAS severity categories^d^			
Baseline			
Marked	2 (50.0)		2 (25.0)
Severe	2 (50.0)		3 (37.5)
Very severe	0		3 (37.5)
Primary endpoint			
Normal	2 (50.0)		5 (71.4)
Moderate	0		1 (12.5)
Marked	2 (50.0)		1 (12.5)
Change baseline to primary endpoint			
No change	1 (25.0)		0
Reduction of one level	1 (25.0)		0
Reduction of two levels	1 (25.0)		4 (57.1)
Reduction of three levels	1 (25.0)		2 (28.6)
Reduction of four levels	0		1 (12.5)
LSAS total score, mean (SD)			
Baseline	83.3 (11.9)		91.8 (15.8)
6-month follow-up	60.0 (17.4)		42.9 (20.4)
Change^b^	− 23.3 (18.0)		− 47.7 (14.7)
*P* value^b^		0.036	

Abbreviations: LSAS, Liebowitz Social Anxiety Scale; *N*, number of participants

^a^Outcomes are based on intent-to-treat set

^b^Change from baseline

^c^*N* = 7 in MDMA group after baseline

^d^Severity categories defined as LSAS total scores ranging from 0 to 54 (normal), 55–65 (moderate), 66–80 (marked), 81–95 (severe), 96–200 (very severe)

**Fig. 2:**
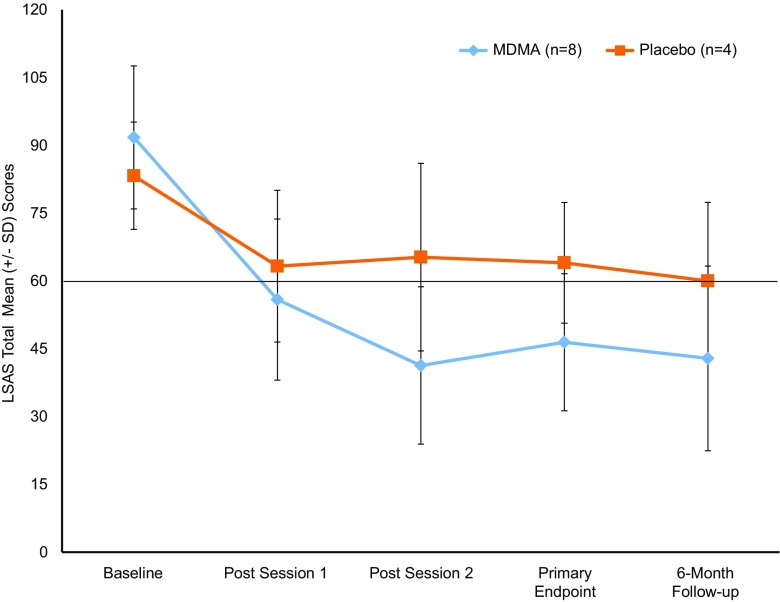
Change over time in LSAS total scores (MDMA *n* = 8 at baseline, *n* = 7 at all other time points; placebo *n* = 4). The primary endpoint occurred 1 month after the second experimental session. The 6-month follow-up visit was 6 months after the primary endpoint. The MDMA group had a greater mean change from baseline than the placebo group at the primary endpoint (*P* = 0.037) and at the 6-month follow-up (*P* = 0.036). The line at LSAS score of 60 represents inclusion criteria minimum score

Reductions were retained for the MDMA group at 6-month follow-up compared to primary endpoint, supporting durability of improvements (MDMA, *t*(6) = 1.117, *P* = 0.307). Alternate definitions of treatment response were explored. The rate of clinical response was defined as a 20-point reduction in LSAS based on prior studies using the LSAS (Simon et al. 2004). The rate of clinically significant changes in SAD symptoms from Baseline was 6/8 (75%) with MDMA versus 2/4 (50%) with placebo. Figure 2 shows a clear linear relationship between visit and mean LSAS score for the MDMA group, whereas no such relationship exists for the placebo group. Changes in secondary/exploratory outcome measures are presented with descriptive statistics. Generally, the results obtained from these measures changed similarly among the groups (Table 1).

### Pharmacodynamic measurements

Consistent with known sympathomimetic effects of MDMA, pharmacodynamic response of blood pressure, pulse, and body temperature (BT) were typically, but not always, elevated in the MDMA group versus placebo (Table 2). Mean peak SBP levels were significantly different between groups (*P* = 0.021). MDMA produced greater elevation in diastolic blood pressure (DBP) than placebo but mean peak DBP values did not significantly differ between groups. SBP values greater than 180 mmHg and DBP above 110 mmHg were not detected. At session end, blood pressure returned to pre-drug levels in both groups, with no clinical intervention. Difference in mean peak pulse rates were significant between groups (*P* = 0.015). Maximum observed pulse was 114 bpm after MDMA. Mean peak temperature was significantly higher in the MDMA group (*P* < 0.001). Maximum BT observed in the MDMA groups was 37.7 °C. At session end, elevation in BT compared to baseline was 0.4 °C in the MDMA group and 0.2 °C in the placebo group, consistent with normal diurnal 0.5 °C increases in the afternoon (Mackowiak et al. 1992). No clinically significant AEs were reported based on elevations in blood pressure, pulse rate, or temperature.

### Safety

No SAEs were reported on this study. No spontaneously reported reactions during experimental sessions were rated as severe. Most commonly reported reactions were anxiety (75.0% MDMA versus 25.0% placebo) and difficulty concentrating (62.5% MDMA versus 25.0% placebo). Fatigue, headache, and sensitivity to cold were also reported (50.0% MDMA versus 0–25.0% placebo). The only severe spontaneously reported reaction was a headache in a participant in the MDMA group on day 1 post-drug. Commonly reported reactions to MDMA were generally mild to moderate, with less frequent reports after the 24-h period following treatment. Reactions were rare after the third day of contact (Table 4).

**Table 4:** Number of participants reporting expected reactions during two MDMA sessions and seven days following

	Placebo (*n* = 4)	MDMA (*n* = 8)
Reactions during experimental sessions, no. (%)^a^		
Anxiety	1 (25.0)	6 (75.0)^b^
Difficulty concentrating	1 (25.0)	5 (62.5)^b^
Fatigue	1 (25.0)	4 (50.0)^b^
Headache	1 (25.0)	4 (50.0)^b^
Lack of appetite	1 (25.0)	3 (37.5)^c^
Muscle tension	1 (25.0)	3 (37.5)
Restlessness	1 (25.0)	3 (37.5)^b^
Sensitivity to cold	0	4 (50.0)
Dizziness	1 (25.0)	1 (12.5)
Low mood	0	2 (28.6)^b^
Perspiration	1 (25.0)	1 (12.5)
Thirst	0	2 (28.6)
Weakness	1 (25.0)	1 (12.5)
Drowsiness	0	1 (12.5)^b^
Impaired gait/balance	0	1 (12.5)^b^
Increased irritability	0	1 (12.5)^b^
Jaw clenching, tight jaw	0	1 (12.5)
Need more sleep	0	1 (12.5)^b^
Ruminations	0	1 (12.5)
None	2 (50.0)	0
Top reactions during 7 days of contact, no. (%)^a^		
Anxiety	1 (25.0)^b^	1 (12.5)
Difficulty concentrating	0	4 (50.0)
Dizziness	1 (25.0)	0
Drowsiness	0	1 (12.5)
Fatigue	2 (50.0)	5 (62.5)^e^
Headache	1 (25.0)	5 (62.5)^b,d^
Increased irritability	1 (25.0)	1 (25.0)
Insomnia	0	1 (12.5)
Jaw clenching, tight jaw	1 (25.0)	0
Lack of appetite	0	3 (37.5)
Low mood	2 (50.0)^c^	4 (50.0)^c^
Need more sleep	2 (50.0)	3 (37.5)^b^
Parasthesias	0	1 (12.5)
Ruminations	1 (25.0)^b^	0
Sensitivity to cold	1 (25.0)	0
Thirst	0	1 (12.5)
Weakness	1 (25.0)	1 (12.5)
None	1 (25.0)	0

Abbreviations: *N*, number of participants

^a^Frequency of subjects who reported an expected, spontaneously reported reaction collected during and 7 days following blinded experimental sessions 1 and 2

^b^One moderate

^c^Two moderate

^d^One severe

^e^Three moderate

Verbatim reports of AEs during the active treatment period were coded via Medical Dictionary for Regulatory Activities (MedDRA V17.1). Most AEs were classified as falling within the overarching class psychiatric disorders (four participants reporting AEs in MDMA group versus three in placebo); none was severe (Table 5). Depressed mood was 25.0% MDMA versus 0.0% after placebo. All AEs were rated mild or moderate (Table 5). Suicidal ideation was the most commonly reported AE; however, prevalence was similar across groups (25.0% both groups) and was pre-existing in medical history. In the MDMA group, AEs rated as moderate based on limitation of daily functions included anxiety, depression, suicidal ideation, and panic attack. In the placebo group, an AE of upper respiratory infection was considered moderate. Instances of positive suicidal ideation occurred during two MDMA sessions and resolved by the following day for two participants. Of these, one participant had a medical history of suicidal behavior, and rates were equivalent between groups; therefore, positive ideation may have been related to the non-drug psychotherapy process.

**Table 5:** Number of participants reporting treatment-emergent psychiatric adverse events

	Placebo (*n* = 4)	MDMA (*n* = 8)
Psychiatric TEAEs, no. (%)^a^		
Anxiety	0	1 (12.5)^b^
Depressed mood	0	2 (25.0)
Depression	1 (25.0)	1 (12.5)^c^
Panic attack	0	1 (12.5)^b^
Panic reaction	0	1 (12.5)
Suicidal ideation	1 (25.0)	2 (25.0)^b^
None	3 (75.0)	4 (50.0)

Abbreviations: TEAEs, treatment emergent adverse events

^a^Frequency of subjects who self-reported psychiatric adverse events after first drug administration until the primary endpoint

^b^One moderate

^c^Two moderate

### Blinding

Participants, therapists, and IR were blinded to drug assignment. Of all 23 experimental sessions, participants incorrectly guessed their treatment assignment in one of eight (12.5%) placebo sessions. One therapist guessed incorrectly in two of eight (25.0%) placebo sessions and two of 15 (13.3%) MDMA sessions; the other therapist guessed incorrectly in one of eight (12.5%) placebo sessions and two of 15 (13.3%) MDMA sessions.

## Discussion

This pilot study is the first to investigate MDMA-assisted psychotherapy to treat generalized social anxiety, which is prevalent and often disabling for autistic adults. At primary endpoint, the mean change from baseline in LSAS scores was significantly greater for the MDMA group compared to the placebo group. The placebo-subtracted effect size for the changes in LSAS from baseline to the primary endpoint and to 6-month follow-up was very large (*d* = 1.4 and 1.1, respectively). Enrollment required a total score of 60 or greater on the LSAS at baseline, in a range highly suggestive of generalized SAD. Scores in this range are typical of individuals entering treatment and indicate high levels of distress and difficulties with social functioning. In addition, high mean scores on both the social anxiety and social avoidance subscales were suggestive of generalized SAD as opposed to specific, focal problems such as public speaking anxiety.

Mean scores for the placebo group improved at primary endpoint, but not to the degree of the MDMA group. In comparison, mean scores for the MDMA group remained below the enrollment cutoff after treatment and continued to decrease during the 5-month period when participants were not receiving therapy. Of seven participants in the MDMA group completing treatment, all dropped two to four levels in severity category, whereas the four participants in the placebo group dropped zero to three levels in severity. In addition, six of seven participants in the MDMA group had a > 20-point drop in LSAS scores compared to two of four participants in the placebo group.

To help mitigate potential bias and to minimize inter-rater variability, the same qualified blinded IR conducted every LSAS administration for all participants, which contributed to a high level of consistency in interview methods and scoring. The IR was not present during experimental sessions and did not discuss clinical impressions with investigators who were present during treatment. Participants were instructed not to inform the IR of beliefs concerning their group assignment during the assessment period. The general impression, supported by spontaneous participant feedback, was that the LSAS was an effective instrument for autistic study participants, who typically prefer quantifying responses without the limitations of multiple choice or Likert scales, which can feel imprecise for respondents.

Participant self-report on subjective effects was congruent with the marked decrease in LSAS mean scores, with no participant reporting a clinically significant increase in social anxiety or avoidance behaviors post-treatment. Examples of changes that were self-reported during audio-recorded post-treatment semi-structured interviews, clinical sessions, and in unstructured correspondence with therapists, included reduced barriers to successful social interactions and increased confidence in school, at work, in friendships, and in romantic relationships. Several participants and SSPs provided accounts of improved interpersonal interactions with family members. Two participants reported being able to initiate dating for the first time, and two reported feeling more comfortable with exploring and expressing gender identity. Examples of participant quotes on subjective effects are included in the Supplemental eTable 7.

The investigators’ clinical impressions regarding the mechanisms of action that made MDMA an effective adjunct to psychotherapy were consistent with research on MDMA’s neurobiological effects. Serotonergic effects likely contributed to previously inaccessible states of calm and well-being most participants reported during MDMA experimental sessions. Several participants experienced increased comfort with prolonged eye-contact and enhanced ability to express emotions verbally. Increases in OT levels after MDMA, as reported in healthy individuals, might have enhanced a sense of connection and enriched therapeutic rapport (Dumont et al. 2009; Hysek et al. 2014; Kuypers et al. 2017). Most participants reported a history of moderate to severe trauma, which is common in the autistic community (Roberts et al. 2015). Studies of MDMA in healthy individuals have demonstrated a reliable reduction of amygdalar activity (Bedi et al. 2009; Carhart-Harris et al. 2014; Carhart-Harris et al. 2015; Gamma et al. 2000) and a perception of less fear (Bedi et al. 2009; Dolder et al. 2018; Hysek et al. 2014), which might have aided participants in our study to remember and process past traumas and engage in corrective emotional experiences that were cathartic during the MDMA experimental sessions. Additional research will be required to determine whether theories of psychophysiological mechanisms of action of MDMA in psychotherapy are generalizable to autistic adult populations.

Investigators did not provide psychoeducation or training on how to implement or improve social skills. However, in the majority of cases, they observed emergence of apparently intact latent social skills (e.g., ease of initiating and sustaining conversation) that manifested and became apparent to observers during experimental sessions with MDMA when participants relaxed. These improvements persisted to varying degrees through follow-up. Eleven of 12 participants reported marked reductions in anxiety responses to in vivo exposure to triggers previously distressing for them, such as making a presentation, speaking on the telephone, entering new social settings, or interacting with authority figures.

One participant who received MDMA (100 and 125 mg) did not show expected changes in BP, HR, or BT and reported no subjective acute effects over the course of treatment. Both investigators present during these two MDMA experimental sessions incorrectly recorded their belief of condition assignment as placebo with high certainty. An ad hoc laboratory analysis after unblinding confirmed the presence of MDMA in a plasma sample taken during an experimental session which ruled out pharmacy or randomization error. This participant stopped taking a prescription SSRI (escitalopram), per protocol, approximately 2 months prior to treatment. Research in clinical settings with diverse study populations on the potential attenuation of effects of MDMA due to genetic factors, prior and recent SSRI use influencing downregulation of serotonin transporters, and other factors specific to autism are indicated as areas of future study.

Psychological function, particularly in regard to expressions of SAD, improved over the 6 months. There were no serious adverse psychological or medically related health events. Although moderate elevations in blood pressure, heart rate, and temperature were observed during most experimental sessions, no participants encountered any acute cardiovascular or hyperthermia crises. Regarding vital signs, there were significant expected elevations in the MDMA group in peak SBP, heart rate, and temperature, but not DBP, and well within margins of safety. BT in the MDMA group remained well within normal range. Long-term follow-up failed to detect any deleterious outcomes. Such findings are consistent with other formally approved MDMA clinical research investigations in people with PTSD (Mithoefer et al. 2018; Mithoefer et al. 2013; Oehen et al. 2013) and healthy controls (Grob et al. 1996; Vizeli and Liechti 2017).

When examining short-term response to treatment (during the experimental sessions and 1 week following), more anxiety (75% of participants) was reported in the MDMA group as compared to the placebo group. Although the protocol did not specify collection of reaction onset time or duration during experimental sessions, we observed that most of the reports reflected transient anxiety within the first hour following MDMA administration, which is common and expected. Virtually all of the adverse effects reported, by both MDMA and placebo participants, were relatively mild and of brief duration. Considerable care was also given to monitoring for emergence of suicidal ideation. The C-SSRS was administered at baseline, during the experimental session, daily for 7 days following each treatment, and at two integrative psychotherapy sessions. While mild levels of suicidal ideation were reported by a few participants, they were evaluated as being of very low risk and were reported at equal frequency (25%) by the MDMA and placebo group. No participants expressed serious suicidal ideation. However, one participant with a history of past suicidal behaviors reported transient suicidal ideation during a personal crisis that quickly resolved.

### Limitations

The small sample size and broad range of scores limits claims about potential impact and generalizability of the treatment, despite the very large effect size for the primary outcome measure. The findings justify the need for future research for treatment of SAD with MDMA-assisted psychotherapy. Furthermore, the sample was too small to compare dose-response effects between subgroups. Heterogeneity in baseline scores and lack of significant differences between groups for the exploratory measures precluded assessment of meaningful clinical response. For example, not all participants presented with clinically significant depression symptoms at baseline, so changes in BDI scores were insignificant even though mean scores dropped below the level of clinical significance after treatment for participants with high baseline BDI scores. This signal supports future studies of MDMA-assisted psychotherapy for depression in autistic adults.

AEs reported to cause mild to moderate limitation of daily function related to depression, anxiety, panic, and suicidal ideation were reported. However, codiagnosis of these psychiatric symptoms and comorbid psychiatric disorders is common in autistic populations, and the sample size was too small for meaningful analysis of trends.

Another limitation was potential for inclusion or exclusion error due to imprecision in available autism diagnosis methods. Standardized assessment by the designated qualified rater with the ADOS-2 (adult module) with scores indicating autism was required for inclusion. However, a more comprehensive assessment would be indicated to confirm a formal diagnosis for some participants with no prior evaluation history.

Effective blinding is a challenge for trials of psychoactive substances when drug effects may be observable to participants and investigators. Delegating all administrations of the LSAS to a blinded IR who never saw the participants during experimental sessions strengthened the blind. One participant and both investigators made incorrect guesses, so double-blinding with an inactive placebo was considered adequate for this study. Both groups in this study received the same type of psychotherapy with encouragement toward self-directed healing and meaning-making. The investigators acknowledge that the MDMA effects that are observable to participants might be a factor that contributes in some way to efficacy of MDMA-assisted therapies.

Investigators had no means to confirm prior abstinence from MDMA, so deception at intake was possible. Undisclosed prior MDMA use had the potential to break the blind for any participant familiar with its effects. In addition, drug screening was completed at baseline and prior to experimental sessions, but undisclosed illicit drug use as well as reported concomitant psychiatric medications during follow-up had the potential to influence 6-month outcomes.

Recruitment delays were a challenge. Autistic adults with SAD experience high levels of social isolation and can be difficult to contact through conventional recruitment methods. Recruitment relied primarily on Internet advertisements, so individuals without online access were less likely to receive information about recruitment. Increasing recruitment through advertisements on drug-interest forums might have increased the likelihood of self-selection bias and subject-expectancy effects. Investigators took steps to mitigate these effects in recruitment, by placing advertisements in online autism forums and engaging in community outreach.

## Conclusions

The two primary goals of this study were to establish feasibility and safety of MDMA-assisted psychotherapy in a controlled clinical setting for SAD in autistic adults; both were successfully established. Changes in LSAS scores and subjective observations were consistent with the hypothesis that anxiety interferes with social functioning in autistic adults and can be alleviated with a combination of MDMA and psychotherapy, supportive preparation, and integrative after care. Findings support more trials of MDMA-assisted psychotherapy in larger samples of adults with SAD.

## Electronic supplementary material

ESM 1 (DOCX 58.7 kb)

ESM 2 (PDF 86.8 kb)
