# Supplementary material for: Reduction in social anxiety after MDMA-assisted psychotherapy with autistic adults: a randomized, double-blind, placebo-controlled pilot study
Source: Psychopharmacology (Berl). 2018 Sep 8;235(11):3137–48. doi: 10.1007/s00213-018-5010-9 (PMC6208958; doi:10.1007/s00213-018-5010-9)
Supplement: Supplementary file 1 — (DOCX 58.7 kb) [file 213_2018_5010_MOESM1_ESM.docx]

**Supplemental Material**

**Article title:** Reduction in social anxiety after MDMA-assisted psychotherapy with autistic adults: A randomized, double-blind, placebo-controlled pilot study

**Journal submitted to**: Psychopharmacology

**Authors**: Alicia L. Danforth PhD, Charles S Grob MD, Christopher Struble MD, Allison Feduccia PhD, Nick Walker MA, Lisa Jerome PhD, Berra Yazar-Klosinski PhD, Amy Emerson BS

**Corresponding author affiliation**: Charles S. Grob, Harbor-UCLA Medical Center, Box 498, 1000 W. Carson Blvd, Torrance, CA 90509. Email: cgrob@labiomed.org

The LSAS is a 24-item, semi-structured interview on the severity of Social Anxiety Disorder. The LSAS has widely been used in clinical studies, including research on social anxiety in autistic adults (Cath *et al.*, 2008, Liebowitz *et al.*, 1985). The LSAS separately assesses fear (0 to 3 = none, mild, moderate, severe) and avoidance (0 to 3 = never, occasionally, often, usually) of 24 social situations, providing an overall social anxiety severity rating and subscale scores for performance fear, performance avoidance, social fear, and social avoidance. The total LSAS score is most routinely used as a primary outcome measure in psychopharmacological trials for treatment of generalized social anxiety disorder. The LSAS has good internal consistency (score inter-correlations ranging from 0.81 to 0.96), reliability, and convergent validity with other measures of anxiety (Fresco *et al.*, 2001, Heimberg *et al.*, 1999). The LSAS was also demonstrated to be sensitive to the effects of pharmacological treatments of social phobia in comparison to placebo (Liebowitz *et al.*, 1992, Liebowitz *et al.*, 2002, Stein *et al.*, 1998) or psychotherapy (Heimberg *et al.*, 1998).

Inclusion/Exclusion Criteria

All participants were required to be age 21 years or older, have at least two years of college education or the equivalent, complete the ADOS-2 Module 4 (adult) (Bastiaansen and al., 2011) by a designated Independent Rater (IR) who was research reliability certified, have moderate to severe symptoms of social anxiety with a combined LSAS score of at least 60. They had to be able to safely discontinue any current psychotropic medications that could interfere with action of the experimental drug. Participants had healthy cardiovascular function, as per electrocardiogram (EKG) and current medical history, and did not suffer from diabetes, glaucoma, seizures, hypertension, liver disease, glaucoma or any other condition that the medical monitor believed might interfere with safe participation in the study. Psychiatric exclusion criteria included family history in first-degree relatives of schizophrenia or bipolar I disorder, or participant diagnoses of active or past psychotic disorder, borderline personality disorder, dissociative identity disorder, eating disorder or active suicidal ideation. Participants affirmed MDMA-naïve status and did not have active substance use disorders. Prior to entry into the study, all participants had a physical examination, EKG, and baseline labs, including urine screening for drugs of abuse and for pregnancy in females. As an additional safety requirement related to the unique psychosocial effects of MDMA, each participant was required to designate a Study Support Partner (SSP), who also signed an informed consent to participate in an adjunctive, non-clinical support role.

**References**

**Bastiaansen, J. & al., e.** (2011). Diagnosing autism spectrum disorders in adults: the use of Autism Diagnostic Observation Schedule (ADOS) module 4. *J Autism Dev Disord* **41**, 1256-66.

**Cath, D. C., Ran, N., Smit, J. H., van Balkom, A. J. & Comijs, H. C.** (2008). Symptom overlap between autism spectrum disorder, generalized social anxiety disorder and obsessive-compulsive disorder in adults: a preliminary case-controlled study. *Psychopathology* **41**, 101-10.

**Fresco, D. M., Coles, M. E., Heimberg, R. G., Liebowitz, M. R., Hami, S., Stein, M. B. & Goetz, D.** (2001). The Liebowitz Social Anxiety Scale: a comparison of the psychometric properties of self-report and clinician-administered formats. *Psychol Med* **31**, 1025-35.

**Heimberg, R. G., Horner, K. J., Juster, H. R., Safren, S. A., Brown, E. J., Schneier, F. R. & Liebowitz, M. R.** (1999). Psychometric properties of the Liebowitz Social Anxiety Scale. *Psychol Med* **29**, 199-212.

**Heimberg, R. G., Liebowitz, M. R., Hope, D. A., Schneier, F. R., Holt, C. S., Welkowitz, L. A., Juster, H. R., Campeas, R., Bruch, M. A., Cloitre, M., Fallon, B. & Klein, D. F.** (1998). Cognitive behavioral group therapy vs phenelzine therapy for social phobia: 12-week outcome. *Arch Gen Psychiatry* **55**, 1133-41.

**Liebowitz, M., Gorman, J., Fyer, A. & Klein, D.** (1985). Social Phobia: Review of a Neglected Anxiety Disorder. *Arch Gen Psychiatry*, 42729-736.

**Liebowitz, M. R., Schneier, F., Campeas, R., Hollander, E., Hatterer, J., Fyer, A., Gorman, J., Papp, L., Davies, S., Gully, R. & et al.** (1992). Phenelzine vs atenolol in social phobia. A placebo-controlled comparison. *Arch Gen Psychiatry* **49**, 290-300.

**Liebowitz, M. R., Stein, M. B., Tancer, M., Carpenter, D., Oakes, R. & Pitts, C. D.** (2002). A randomized, double-blind, fixed-dose comparison of paroxetine and placebo in the treatment of generalized social anxiety disorder. *J Clin Psychiatry* **63**, 66-74.

**Stein, M. B., Liebowitz, M. R., Lydiard, R. B., Pitts, C. D., Bushnell, W. & Gergel, I.** (1998). Paroxetine treatment of generalized social phobia (social anxiety disorder): a randomized controlled trial. *JAMA* **280**, 708-13.

eTable 1. Secondary Outcome Measures^a^

|  | **Placebo**  **(n = 4)** | **MDMA**  **(n = 8)** |
| --- | --- | --- |
| BDI-II Total Score, mean (SD) |  |  |
| Baseline | 17.0 (16.5) | 16.3 (12.6) |
| Primary Endpoint | 3.8 (7.5) | 6.3 (6.4) |
| Change^b^ | -13.3 (12.2) | -10.1 (13.1) |
| 6-month follow-up | 10.5 (11.6) | 6.6 (4.9) |
| ERQ Reappraisal Total Score, mean (SD) |  |  |
| Baseline | 28.5 (4.7) | 24.8 (4.8) |
| Primary Endpoint | 27.3 (8.6) | 26.3 (5.5) |
| Change^b^ | -1.3 (6.8) | 2.0 (8.7) |
| 6-month follow-up | 31.5 (7.4) | 30.3 (7.1) |
| ERQ Suppression Total Score, mean (SD) |  |  |
| Baseline | 18.3 (4.9) | 19.1 (4.8) |
| Primary Endpoint | 15.0 (3.7) | 18.0 (4.6) |
| Change^b^ | -3.3 (4.0) | -1.7 (6.5) |
| 6-month follow-up | 15.5 (4.9) | 13.1 (5.4) |
| IRI Perspective Taking Score, mean (SD) |  |  |
| Baseline | 20.0 (4.7) | 15.0 (7.4) |
| Primary Endpoint | 14.0 (4.2) | 17.8 (5.9) |
| Change^b^ | -6.0 (8.6) | 1.7 (4.7) |
| 6-month follow-up | 19.8 (3.6) | 18.6 (4.1) |
| IRI Fantasy Score, mean (SD) |  |  |
| Baseline | 18.0 (8.8) | 17.8 (6.1) |
| Primary Endpoint | 21.0 (5.9) | 17.7 (6.3) |
| Change^b^ | 3.0 (4.4) | 0.1 (4.2) |
| 6-month follow-up | 21.0 (6.4) | 17.0 (6.7) |
| IRI Empathic Concern Score, mean (SD) |  |  |
| Baseline | 18.8 (8.1) | 19.8 (4.8) |
| Primary Endpoint | 19.3 (8.4) | 22.3 (3.7) |
| Change^b^ | 0.5 (3.0) | 2.0 (3.2) |
| 6-month follow-up | 17.5 (9.3) | 22.0 (3.8) |
| IRI Personal Distress Score, mean (SD) |  |  |
| Baseline | 16.5 (6.8) | 12.5 (5.2) |
| Primary Endpoint | 12.3 (7.6) | 9.0 (4.4) |
| Change^b^ | -4.3 (2.6) | -2.3 (5.0) |
| 6-month follow-up | 13.3 (9.1) | 6.6 (3.7) |
| QOLQ Total Score, mean (SD) |  |  |
| Baseline | 72.8 (13.3) | 78.3 (9.2) |
| 6-month follow-up | 82.0 (27.0) | 89.9 (8.6) |
| Change^c^ | 9.3 (18.1) | 10.0 (13.0) |
| RSES Total Score, mean (SD) |  |  |
| Baseline | 14.5 (4.4) | 14.1 (6.9) |
| Primary Endpoint | 19.8 (6.8) | 20.0 (5.5) |
| Change^b^ | 5.3 (4.9) | 4.6 (6.8) |
| 6-month follow-up | 20.8 (7.8) | 21.6 (5.5) |
| STAI Trait Score, mean (SD) |  |  |
| Baseline | 50.8 (18.6) | 58.8 (11.0) |
| Primary Endpoint | 39.0 (15.9) | 42.6 (13.5) |
| Change^b^ | -11.8 (17.2) | -15.0 (13.8) |
| 6-month follow-up | 38.3 (18.5) | 40.9 (12.5) |
| STAI State Score, mean (SD) |  |  |
| Baseline | 44.8 (18.6) | 48.5 (16.4) |
| Primary Endpoint | 30.5 (15.2) | 35.9 (11.1) |
| Change^b^ | -14.3 (12.2) | -11.0 (17.9) |
| 6-month follow-up | 26.0 (6.4) | 37.4 (15.7) |
| TAS20 Total Score, mean (SD) |  |  |
| Baseline | 52.0 (15.4) | 54.6 (8.9) |
| Primary Endpoint | 48.0 (11.9) | 45.6 (7.3) |
| Change^b^ | -4.0 (19.4) | -9.6 (5.3) |
| 6-month follow-up | 37.3 (15.2) | 45.7 (9.5) |
| TASIT Minimal Score, mean (SD) |  |  |
| Baseline | 50.8 (8.4) | 52.5 (7.3) |
| Primary Endpoint | 47.0 (14.7) | 53.6 (5.7) |
| Change^b^ | -3.8 (10.7) | 1.4 (8.3) |
| 6-month follow-up | 54.5 (11.0) | 57.0 (2.9) |
| TASIT Enriched Score, mean (SD) |  |  |
| Baseline | 55.5 (5.4) | 53.6 (5.7) |
| Primary Endpoint | 49.3 (9.0) | 55.1 (4.9) |
| Change^b^ | -6.3 (5.0) | 2.3 (3.1) |
| 6-month follow-up | 53.5 (9.7) | 56.1 (3.7) |
| PSS Total Score, mean (SD) |  |  |
| Baseline | 18.0 (11.0) | 25.0 (8.6) |
| Primary Endpoint | 16.3 (12.0) | 17.1 (9.2) |
| Change^b^ | -1.8 (7.5) | -7.4 (13.2) |
| 6-month follow-up | 15.5 (10.4) | 15.9 (8.7) |

^a^ Outcomes are based on intent-to-treat set

^b^ Change from Baseline

^c^ Change from Baseline to 6-month follow-up (measure was not given at the Primary Endpoint)

eTable 2. Vital Signs during Experimental Sessions

|  |  | **Placebo**  **(n = 4)** | **MDMA**  **(n = 8)** |
| --- | --- | --- | --- |
| **Systolic Blood Pressure (mmHg)** | | | |
| Pre-drug  Mean (SD)  Min/Max |  | 131.0 (11.6)  112/144 | 114.8 (11.3)  92/137 |
| Peak  Mean (SD)  Min/Max |  | 142.5 (11.8)  126/159 | 125.9 (16.6)^a^  110/174 |
| Final  Mean (SD)  Min/Max |  | 126.9 (5.6)  121/138 | 116.4 (9.2)  105/135 |
| **Diastolic Blood Pressure (mmHg)** | | | |
| Pre-drug  Mean (SD)  Min/Max |  | 76.9 (10.0)  64/89 | 64.7 (7.1)  52/81 |
| Peak  Mean (SD)  Min/Max |  | 82.3 (11.2)  72/106 | 78.1 (7.4)  62/89 |
| Final  Mean (SD)  Min/Max |  | 74.5 (8.6)  62/88 | 67.9 (6.2)  59/82 |
| **Heart Rate (bpm)** | | | |
| Pre-drug  Mean (SD)  Min/Max |  | 57.3 (9.7)  47/78 | 71.7 (10.8)  53/88 |
| Peak  Mean (SD)  Min/Max |  | 75.3 (10.3)  63/94 | 90.2 (14.1)^a^  71/114 |
| Final  Mean (SD)  Min/Max |  | 67.4 (7.4)  59/83 | 80.4 (13.8)  58/101 |
| **Body Temperature (°C)** | | | |
| Pre-drug  Mean (SD)  Min/Max |  | 36.5 (0.2)  36.1/36.8 | 36.7 (0.5)  35.9/37.4 |
| Peak  Mean (SD)  Min/Max |  | 36.8 (0.1)  36.6/36.9 | 37.3 (0.3)^b^  36.7/37.7 |
| Final  Mean (SD)  Min/Max |  | 36.7 (0.2)  36.4/36.9 | 37.1 (0.3)  36.6/37.6 |

Abbreviations: Min, minimum; Max, maximum; mmHg, millimeters of mercury; °C, Celsius; bpm, beats per minute

^a^ P < 0.05

^b^ P < 0.001

eTable 3. Results of the Columbia Suicide Severity Rating Scale (C-SSRS)^a^ across Experimental Sessions

| **Experimental Session 1**  **No. (%)** | | | | | | | | |
| --- | --- | --- | --- | --- | --- | --- | --- | --- |
|  | | Pre-  Drug^b^ | During-  Drug^c^ | Integration Visit 1 | Contact Day 2 | Contact Day 7 | Integration Visit 2 | Integration Visit 3 |
| Placebo | PI  SI  PB  N | 0 (0)  0 (0)  0 (0)  4 | 0 (0)  0 (0)  0 (0)  4 | 0 (0)  0 (0)  0 (0)  4 | 0 (0)  0 (0)  0 (0)  4 | 0 (0)  0 (0)  0 (0)  3 | 0 (0)  0 (0)  0 (0)  4 | 0 (0)  0 (0)  0 (0)  4 |
| MDMA | PI  SI  PB  N | 0 (0)  0 (0)  0 (0)  8 | 1 (14.3)  0 (0)  0 (0)  7 | 1 (12.5)  0 (0)  0 (0)  8 | 0 (0)  0 (0)  0 (0)  8 | 0 (0)  0 (0)  0 (0)  8 | 1 (12.5)  0 (0)  0 (0)  8 | 2 (28.6)  0 (0)  0 (0)  7 |
| **Experimental Session 2**  **No. (%)** | | | | | | | | |
| Placebo | PI  SI  PB  N | 0 (0)  0 (0)  0 (0)  3 | 0 (0)  0 (0)  0 (0)  3 | 0 (0)  0 (0)  0 (0)  4 | 0 (0)  0 (0)  0 (0)  4 | 0 (0)  0 (0)  0 (0)  4 | 0 (0)  0 (0)  0 (0)  4 | 1 (25.0)  0 (0)  0 (0)  4 |
| MDMA | PI  SI  PB  N | 0 (0)  0 (0)  0 (0)  8 | 0 (0)  0 (0)  0 (0)  7 | 0 (0)  0 (0)  0 (0)  7 | 0 (0)  0 (0)  0 (0)  7 | 0 (0)  0 (0)  0 (0)  7 | 0 (0)  0 (0)  0 (0)  7 | 2 (28.6)  0 (0)  0 (0)  7 |

Abbreviations: PI, Positive Ideation; SI, Serious Ideation; PB, Positive Behavior; N, Number of Participants

^a^ According to the C-SSRS scoring guide, scores of four or five on the suicidal ideation category are considered serious ideation, and scores of one or greater are considered positive behavior or ideation.

eTable 4. Results of the Columbia Suicide Severity Rating Scale (C-SSRS)^a^ at 6-month Follow-up

|  | **Placebo**  **(n = 4)** | **MDMA**  **(n = 7)** |
| --- | --- | --- |
| 6-month Follow-up C-SSRS, No. (%) |  |  |
| Positive Ideation | 2 (50.0) | 0 |
| Serious Ideation | 0 | 0 |
| Positive Behavior | 0 | 0 |

Abbreviations: PI, Positive Ideation; SI, Serious Ideation; PB, Positive Behavior

^a^ According to the C-SSRS scoring guide, scores of four or five on the suicidal ideation category are considered serious ideation, and scores of one or greater are considered positive behavior or ideation.

eTable 5. Number of Participants Reporting Treatment Emergent Adverse Events through the Primary Endpoint

|  | **Placebo**  **(n = 4)** | **MDMA**  **(n = 8)** |
| --- | --- | --- |
| Gastrointestinal disorders | 0 | 0 |
| Infections and infestations | 1 (25.0)^a^ | 1 (12.5) |
| Injury, poisoning and procedural complications | 0 | 1 (12.5) |
| Nervous system disorders | 0 | 1 (12.5)^a^ |
| Psychiatric Disorders | 0 | 4 (50.0)^b^ |
| Reproductive systems | 0 | 1 (12.5)^a^ |
| Respiratory, thoracic and mediastinal disorders | 0 | 1 (12.5) |
| None | 3 (75.0) | 4 (50.0) |

^a^ One moderate

^b^ Two moderate

eTable 6. Number of Participants Reporting Adverse Events after the Primary Endpoint to 6-month Follow-up

|  | **Placebo**  **(n = 4)** | **MDMA**  **(n = 7)** |
| --- | --- | --- |
| Gastrointestinal disorders | 1 (25.0) | 0 |
| General disorders and administration site conditions | 0 | 1 (14.3)^a^ |
| Infections and infestations | 0 | 1 (14.3) |
| Injury, poisoning and procedural complications | 0 | 1 (14.3) |
| Nervous system disorders | 0 | 1 (14.3)^a^ |
| Psychiatric Disorders | 1 (25.0) | 0 |
| None | 3 (75.0) | 4 (57.1) |

^a^ One moderate
