# Supplementary material for: Reduction in social anxiety after MDMA-assisted psychotherapy with autistic adults: a randomized, double-blind, placebo-controlled pilot study
Source: Psychopharmacology (Berl). 2018 Sep 8;235(11):3137–48. doi: 10.1007/s00213-018-5010-9 (PMC6208958; doi:10.1007/s00213-018-5010-9)
Supplement: Supplementary file 2 — (PDF 86.8 kb) [file 213_2018_5010_MOESM2_ESM.pdf]

eTable 7

Examples of Participant Statements About Results of MDMA-Assisted Therapy

Participant	Months Post-Treatment	Statement
AA	4.5	"I feel optimistic and can better empathize with others."[a]
BB	6	"I felt like I was experiencing my best self and seeing the world for the first time and seeing myself for the first time."[b]
CC	9.5	"Being a participant in the study affected me in many ways. My self esteem [sic] increased, my social anxiety decreased, love flooded in, my heart healed, and I'm more resilient."[a]
CC	9.5	". . . I have been able to sustain flirtatious conversations for a longer time. In these conversations, I realized communication is not just about talking. Now, I take time to notice my emotions and others [sic] emotions before talking."[a]
DD (SPP)	20.5	"Just wanted you to know that G. is really doing well. He is so warm, relaxed, and confident now. He even talks to his instructors, something he would never do in the past."[c]
EE	27	"I am certain that I would be much worse off today if didn't go through that experience. . . I am making good progress toward a better, healthier life."[a]
FF	27.5	"One main thing that I have taken away from the sessions, but only now realizing it, is that I am now more 'me'. If people like the true me for who I am that is wonderful. If not, I am much less thin skinned [sic] and allow myself to be more accepting of it,"[a]

[a] *ad hoc* correspondence from participant
[b] audio-recorded qualitative interview
[c] *ad hoc* correspondence from Study Support Partner (SSP)
